# Unrecognized myocardial infarction detected on cardiac magnetic resonance imaging: Association with coronary artery calcium score and cardiovascular risk prediction scores in asymptomatic Asian cohort

**DOI:** 10.1371/journal.pone.0204040

**Published:** 2018-09-14

**Authors:** Min Jae Cha, Sung Mok Kim, Yiseul Kim, Hyun Su Kim, Soo Jin Cho, Jidong Sung, Yeon Hyeon Choe

**Affiliations:** 1 Department of Radiology, Samsung Medical Center, Sungkyunkwan University School of Medicine, Gangnam-gu, Seoul, Republic of Korea; 2 Cardiovascular Imaging Center, Heart Vascular and Stroke Institute, Samsung Medical Center, Gangnam-gu, Seoul, Republic of Korea; 3 Center for Health Promotion, Samsung Medical Center, Gangnam-gu, Seoul, Republic of Korea; 4 Division of Cardiology, Department of Medicine, Sungkyunkwan University School of Medicine, Prevention & Rehabilitation Center, Heart Vascular & Stroke Institute, Samsung Medical Center, Seoul, Republic of Korea; Scuola Superiore Sant'Anna, ITALY

## Abstract

**Background:**

To investigate the association between unrecognized myocardial infarction (UMI) assessed with cardiac magnetic resonance (CMR) and coronary artery calcium (CAC) and cardiovascular risk prediction scores in asymptomatic Asian subjects.

**Materials and methods:**

Total 872 asymptomatic subjects without prior cardiovascular event (male:female, 817:55; age, 53.88 ± 5.91) who underwent both CMR and CAC scoring CT were included. UMI were accessed and framingham risk score (FRS) and ASCVD (atherosclerotic cardiovascular disease) risk score by ACC/AHA were calculated.

**Results:**

Late gadolinium enhancement indicating UMI was noted in 23 of 872 subjects (2.64%), but only three of them showed ECG abnormality (13.04%). Subjects with UMI showed higher CAC scores, FRS, and ASCVD scores than those without UMI (*p* < .001, *p =* .011 and *p =* .024, respectively). The prevalence of UMI differed significantly according to the CAC scores as follows: 1% in CAC = 0 (4/403), 1% in 1 ≤ CAC <100 (2/293), 6.1% in 100 ≤ CAC < 400 (7/114) and 14.5% in CAC ≥ 400 (9/62), respectively (*p* < .001). Receiver operating characteristics (ROC) analysis by using CAC score demonstrated an area under the curve (AUC) of 0.816 (95% confidence interval (CI), 0.780–0.848; *p* < .0001) for predicting UMI, which is superior to FRS [AUC, 0.712; 95% CI, 0.671–0.751; *p* = .009] and ASCVD risk score [AUC, 0.689; 95% CI, 0.648–0.729; *p* = .036].

**Conclusion:**

The prevalence of UMI increases with increasing burden of CAC and FRS. CAC score is a good discriminator for UMI, superior to FRS and ASCVD score, in asymptomatic population.

## Introduction

Introduction of late gadolinium enhancement (LGE) images on cardiac magnetic resonance (CMR) allows accurate detection of myocardial infarctions (MIs) and other myocardial scars. With advances of LGE imaging technique, the detection rate of clinically unrecognized MIs (UMIs) has increased. Earlier studies demonstrated that CMR depicts significantly larger number of UMIs than has previously been estimated by electrocardiography (ECG).[1–3]

Until now, many researchers have sought for the clinical implication of UMIs detected on the CMR. Various population studies have been performed in Sweden, Iceland, Scotland and US to document the prevalence and clinical impact of myocardial scars and UMIs.[1,3–6] Studies including patients with coronary artery disease (CAD) stated that the presence of UMI is an independent predictor of major adverse cardiac events (MACE) and cardiac mortality.[2,7,8] Several studies of elderly population also showed that the presence of UMI entailed a risk for MACE.[9,10] In addition, there were studies on the clinical significance of UMI based on highly selected patients such as those with diabetes mellitus or aortic stenosis.[11–13]

The prevalence of UMIs has been described varying 7.8~25%.[1–3,8,9] However, previous studies might have inflated the prevalence of UMIs in general population, because they were performed in elderly populations or included those with known CAD and specific cardiovascular risk factors. The prevalence of UMIs in asymptomatic general population without prior cardiovascular event has not been thoroughly studied, especially in Asian population. In addition, there is little information on the association between UMI depicted on CMR and known risk stratification methods such as Framingham risk score (FRS) and ASCVD (atherosclerotic cardiovascular disease) risk score proposed by American College of Cardiology/American Heart Association (ACC/AHA) and coronary artery calcium (CAC) score. Thus, the aim of this study is to investigate the association between UMIs and CAC score and cardiovascular risk prediction scores to identify diagnostic accuracy for UMI prevalence in asymptomatic Asian individuals.

## Materials and methods

The institutional review board of Samsung medical center approved the study (IRB-2018-05-187), and informed consent was waived for the use of patients’ medical and imaging data.

### Study population

**Fig 1:**
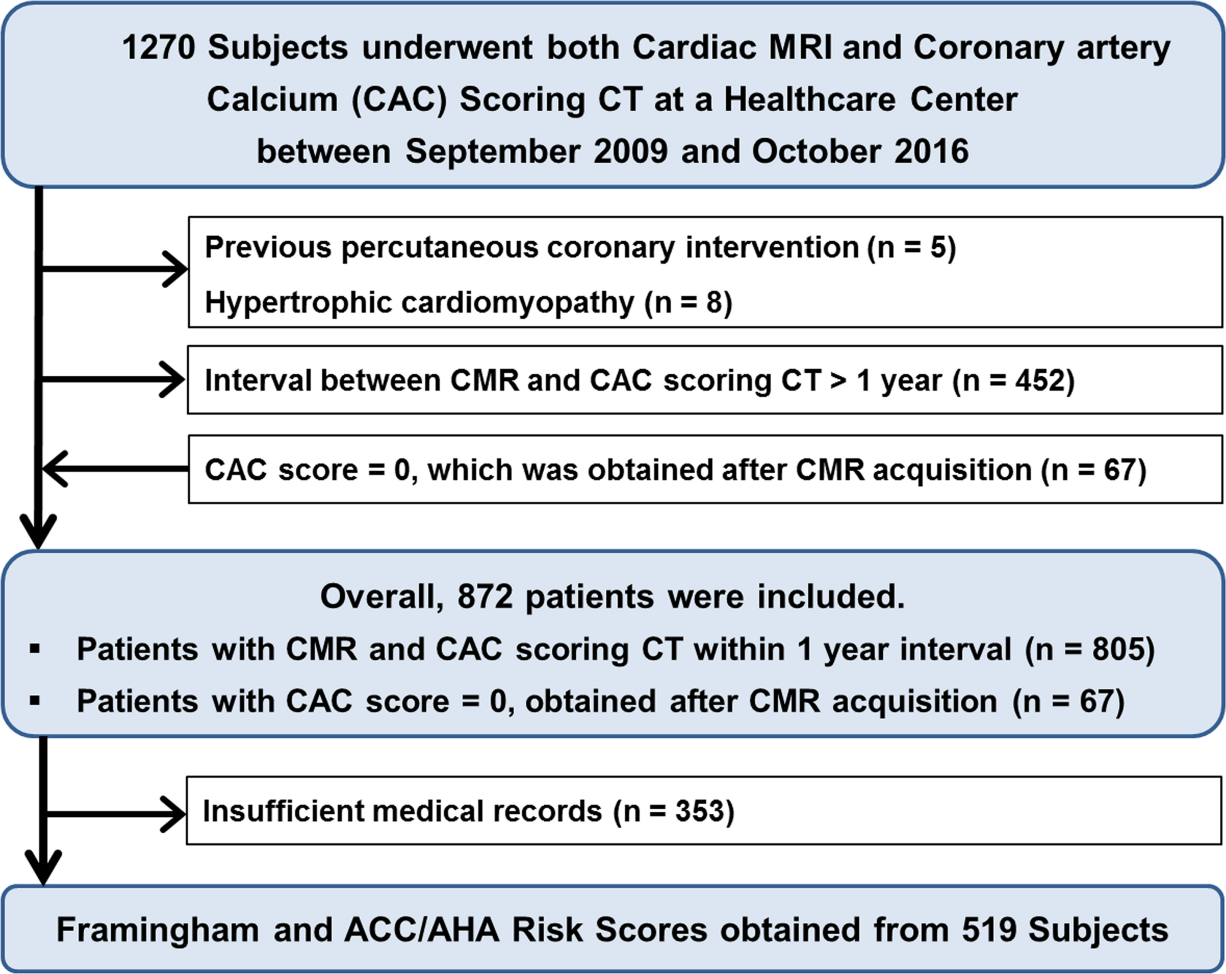
Composition of the study subjects.

We identified 1270 asymptomatic subjects over 40 years of age who underwent both CMR and coronary artery calcium scoring CT for a health checkup at the Health Promotion Center of Samsung Medical Center between September 2009 and October 2016. Among them, 465 patients were excluded for the following reasons: 1) history of percutaneous transluminal coronary angioplasty due to obstructive CAD (n = 5), 2) nonischemic cardiomyopathy such as hypertrophic cardiomyopathy confirmed on CMR (n = 8), and 3) Interval between acquisition of CMR and CAC scoring CT longer than a year (n = 452). Through medical chart review, we confirmed that none of the study population had visited hospital or medicated for chest pain or chest discomfort in the past. In addition, none had previous history of cardiac operation or diagnosed with congenital heart disease or other nonischemic cardiomyopathy. Sixty-seven patients whose CAC score was 0, which was obtained more than a year after CMR acquisition, were additionally included, under the assumption that their CAC score was 0 at the time of CMR acquisition. Finally, a total of 872 self-referred asymptomatic subjects (male:female, 817:55; age, 53.88 ± 5.91) were included in our study (Fig 1).

The clinical information and laboratory results were obtained from chart review. Cardiovascular risk scores were calculated in 519 of 872 subjects, as full medication history of 353 subjects was not available due to insufficient medical records. Ten-year risk of cardiovascular event was calculated based on FRS, and subjects were classified into low risk (10% or less risk at 10 years), intermediate risk (10–20%) and high risk (20% or more).[14] ASCVD risk scores were also assessed based on the ACC/AHA guideline published on 2013.[15] ECG abnormality was defined as presence of pathologic Q-wave, ischemic ST-segment or T-wave changes, or complete left bundle branch block (LBBB) on a resting 12-lead ECG obtained on the day of CMR acquisition.

### CMR acquisition

All subjects underwent CMR in a 1.5-T scanner (Magnetom Avanto, Syngo MR B17 version; Siemens Medical Solutions, Erlangen, Germany) with a 32-channel phased-array receiver coil. CMR scans consisted of localizing images (axial, coronal, and sagittal), cine scans (2-chamber view, 3-chamber view, 4-chamber view, and short-axis view), and LGE scans. LGE imaging was acquired using a phase-sensitive inversion recovery (PSIR) technique 15 min after injection of 0.2 mmol/kg gadobutrol (Gadovist; Bayer Healthcare, Berlin, Germany) at an injection rate of 3 ml/sec, followed by a 30-ml saline flush. Contiguous short-axis image acquisition of 10–12 slices at 6 mm thickness and a 4-mm interslice gap was used. Inversion delay times were typically 280–360 msec. Detailed CMR protocol has been described elsewhere.[16]

### CAC score acquisition

All CT scans were performed using a 64-slice scanner system (Lightspeed, GE Healthcare, Waukesha, WI, USA) and a 40-slice scanner system (Brilliance 40, Philips, Hamburg, Germany). Tube voltage was 120 kVp and tube current was 125 mA. Step and shoot mode was used with prospectively ECG triggered to 75% of the R-R interval in subjects with a heart rate (HR) at most 65 beats per minute (bpm) and 45% of the R-R interval in subjects with a HR faster than 65 bpm. Imaging was reconstructed into a 2.5-mm slice thickness with a 512 X 512 matrix and a 25-cm field-of-view. No premedication with nitrate or beta-blocker was administered. CAC score analysis was performed using dedicated software (Terarecon Aquarius Workstation, San Mateo, California, USA) and CAC scores were subsequently calculated using the methods described by Agatston et al..[17] Then, subjects were classified into four groups according to CAC score as follows: 0, 1–99, 100–399 and ≥ 400.[3,18]

### Image analysis

CMR data were analyzed using a commercially available dedicated software tool (Dynamic Signal Analysis, Argus, Siemens Medical Solutions) by an experienced MR technician (Y.K., with 6 year experience), who was blinded to the clinical information of the participants. Parameters such as left ventricular (LV) end-diastolic volume, LV end-systolic volume, LV ejection fraction, LV mass, and stroke volume (SV) were obtained by drawing LV contours at the end of diastole and systole. Papillary muscles and trabeculation were included in the LV cavity for volume and mass measurements. Cardiac output was calculated as the product of SV and HR. The LV mass index (LVMI) and cardiac index were calculated as LV mass and cardiac output divided by the body surface area, respectively.

**Fig 2:**
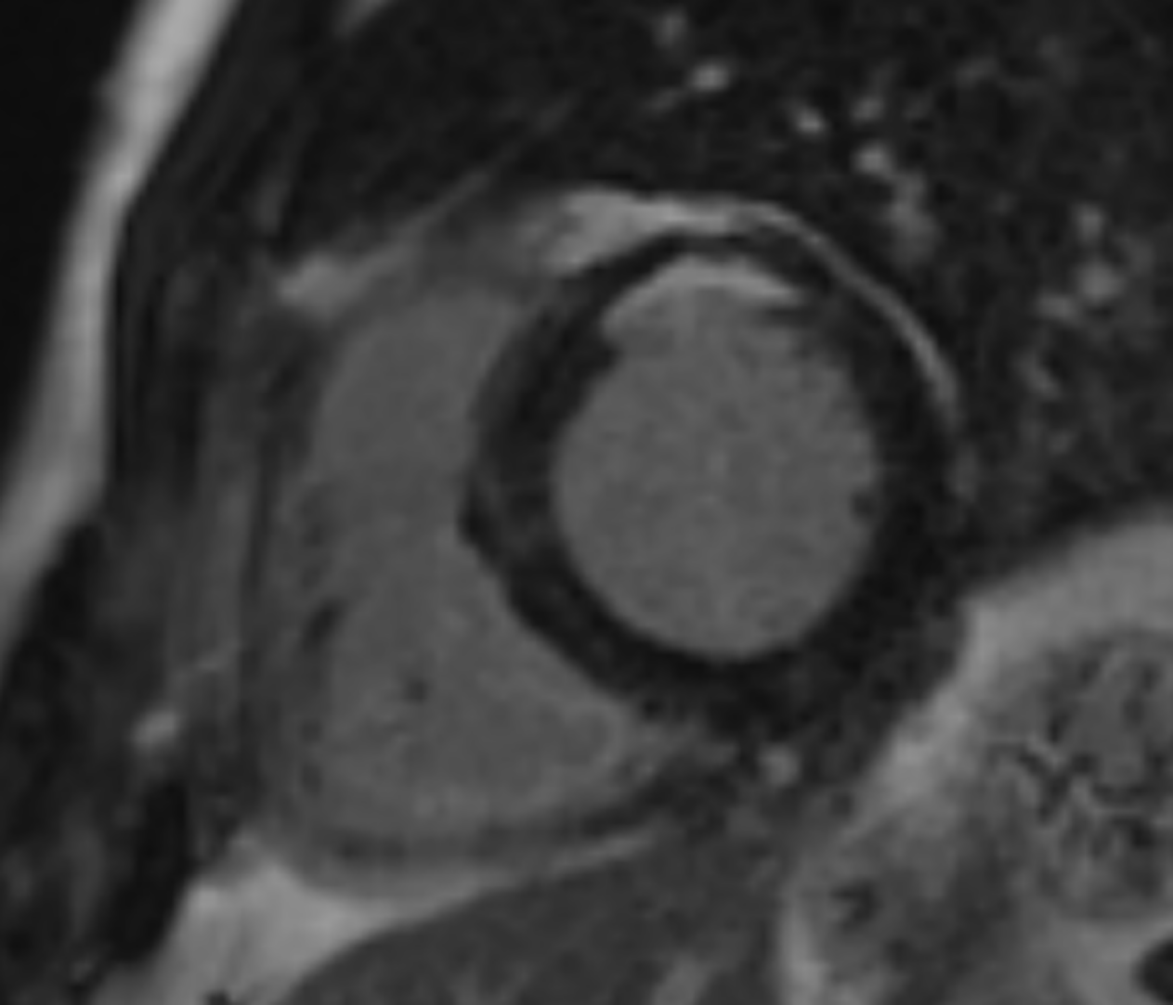
Unrecognized myocardial infarction as detected with cardiac magnetic resonance imaging in a 58-year-old male. Late gadolinium enhancement image demonstrated subendocardial hyperenhancement at anterior wall of left ventricular base level. His coronary artery calcium score was 462.

Analysis of the LGE sequences was performed by use of picture archiving and communication system (Centricity 3.0; GE Healthcare, Mt. Prospect, IL, USA). The presence of LGE was interpreted by the consensus of two observers (M.J.C., with 4-year experience and S.M.K., with 7-year experience in cardiovascular imaging) blinded to patient history. High signal-intensity lesion with subendocardial or transmural involvement, located in territories consistent of specific epicardial coronary arteries, on LGE images were considered to represent UMIs (Fig 2). The location and extent of UMI was evaluated using the American Heart Association (AHA) 17 segment model.[19]

### Statistical analysis

Continuous data were expressed as mean and standard deviation, and all categorical data were presented as proportions. Student’s t-test and one-way ANOVA were used to test for differences in normally distributed continuous variables, and the Wilcoxon rank sum test, Mann-whitney test, and Kruskal-Wallis test were used for comparison involving variables that were not normally distributed. The Jonckheere-Terpstra test was used to report the association of variables according to the groups classified with CAC score and FRS. Categorical variables were compared with the chi-square test or Fisher’s exact test, as appropriate. A two-tailed *p* values less than 0.05 were considered statistically significant. The confidence level and cutoff value of each variable was analyzed with use of the receiver operating characteristic (ROC) method. Statistical analysis was executed using SAS version 9.4 (SAS Institute, Cary, NC).

## Results

### Prevalence UMI

Among 872 subjects, LGE indicating UMI was noted in 23 patients (2.64%). In terms of elderly population, the prevalence of UMI was elevated to 3.74% (4/107) for subjects older than 60 and 4.55% (2/44) for those older than 65. Only three of 23 patients showed ECG abnormality (Q-wave, ischemic ST-segment changes or complete LBBB), with a sensitivity of 13.04% for detecting MI. Among 23 patients with UMI, 15 showed focal MI involving only one segment, whereas remaining eight patients showed MI involving multiple segments, ranging from three to 10 segments. In terms of three patients who showed ECG abnormality, the infarcted myocardial territory was significantly larger than those without ECG abnormality (8.67 ± 1.53 segments vs. 1.95 ± 1.88 segments; *p =* .006), and all three of them showed transmural extent of infarction of over 75%. The coronary territories of UMIs were as follows: 11 patients with left anterior descending artery (LAD)-territory, two patients with left circumflex coronary artery (LCX)-territory, five patients with right coronary artery (RCA)-territory, one patient with LAD+LCX-territories, two patients with LAD+RCA-territories, and two patients with LAD+LCx+RCA-territories.

**Fig 3:**
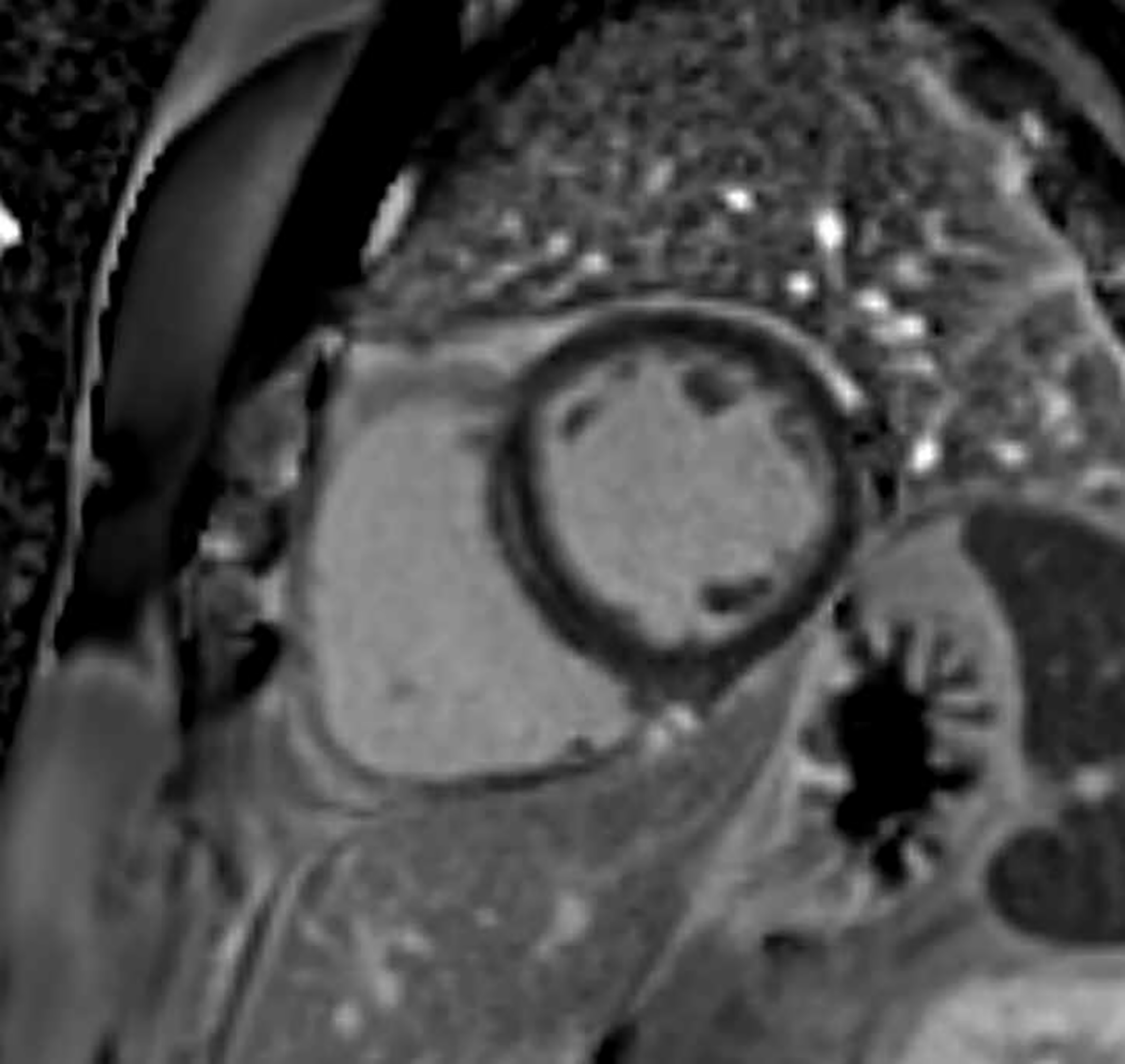
Atypical pattern of late gadolinium enhancement detected with cardiac magnetic resonance imaging in a 50-year-old male. Linear mid-myocardial wall enhancement is noted in mid to basal interventricular septum. His coronary artery calcium score was 0.

In 12 of 872 (1.38%), atypical pattern LGE, not typical for MI, showing nonspecific epicardial or mid-wall LGE without relation to a coronary territory on CMR was noted (Fig 3). The most common feature of these atypical LGE was linear mid-wall enhancement (9 of 12 subjects), followed by focal myocardial enhancement at RV insertion point (4 of 12 subjects).

In terms of CMR parameters such as LV function, reduced LVEF below 55% (asymptomatic mild LV dysfunction) was observed in 20 of 872 (2.3%) subjects, with a range from 45.7% to 54.8%. However, only one of them had UMI and none of them showed atypical LGE.

### Comparison of cohort characteristics and risk stratification scores according to the presence of UMI

The baseline characteristics and CMR parameters of the patients with and without UMI are summarized in Table 1. Subjects with UMI showed higher serum NT-proBNP and glucose level than those without UMI (*p* < .001 and *p* = .030, respectively). In addition, CAC score was significantly higher in subjects with UMI (*p <* .001). In terms of LV parameters on CMR, however, LV function and myocardial mass did not differ significantly between the two groups.

**Table 1:** Comparison of cohort characteristics between those with and without late gadolinium enhancement.

Parameters	No LGE (n = 849)	UMI (n = 23)	*P value*
Male (%)	794 (93.52%)	23 (100%)	*0*.*391*
Age (years)	53 (50–53)	54 (52–59)	*0*.*098*
BMI (kg/m^2^)	24.8 (23.2–26.6)	25.1 (22.4–26.9)	*0*.*746*
ECG abnormality (%)	11 (1.30%)	3 (13.04%)	***0*.*005***
NT-proBNP (pg/mL)	20.3 (11.3–35.4)	33.8 (14.0–69.9)	***< 0*.*001***
Systolic Blood Pressure (mm Hg)	119 (110–130)	122 (111–132)	*0*.*441*
Diastolic Blood Pressure (mm Hg)	77 (71–84)	80 (66–85)	*0*.*608*
Total cholesterol level (mg/dL)	192 (168–218)	186 (158–209)	*0*.*317*
Triglyceride (mg/dL)	119.5 (89.3–170.5)	111 (72–138)	*0*.*820*
HDL cholesterol level (mg/dL)	52 (44–61)	51 (39–74)	*0*.*360*
LDL cholesterol level (mg/dL)	123 (100–145)	105 (79–138)	*0*.*149*
Plasma Glucose level (mg/dL)	98 (91–105)	100 (93–117)	***0*.*030***
CAC Score	4 (0–57)	337 (81–796)	***< 0*.*001***
LVEDV, ml	129.6 (116.8–146.0)	128.5 (98.2–148.3)	*0*.*822*
LVESV, ml	43.8 (36.5–51.9)	47.2 (30.8–52.9)	*0*.*856*
LVEF, %	66.3 (62.2–69.9)	65.9 (60.5–68.9)	*0*.*714*
LVSV, ml	86.2 (76.2–95.7)	84.6 (67.4–92.4)	*0*.*568*
Cardiac index, l/min/m^2^	3.0 (2.8–3.4)	2.9 (2.7–3.1)	*0*.*612*
LVMI, g/m^2^	58.9 (52.3–66.1)	63.4 (56.4–70.5)	*0*.*085*

Note. Median (interquartile range), LGE = late gadolinium enhancement, UMI = unrecognized myocardial infarction, BMI = body mass index, CAC = coronary artery calcium, LVEDV = left ventricular end-diastolic volume, LVESV = left ventricular end-systolic volume, LVEF = left ventricular ejection fraction, LVSV = left ventricular stroke volume, LVMI = left ventricular mass index

In the comparison between one-segment (n = 15) and multi-segment UMI (n = 8), CAC score was significantly higher in multi-segment UMI group [median (interquartile), 1043.5 (447.5–2346.3) vs. 154 (10–387), *p =* .011]. However, there was no significant difference in CMR parameters such as LV function and LV mass. When we compared transmural (n = 4) and non-transmural (n = 19) infractions among UMI patients, LVEF was significantly reduced in those with transmural UMI [median (interquartile), 59.9 (55.7–62.1) vs. 67.8(61.3–70.7), *p =* .032]. When we compared CAC scores of each coronary artery according to the presence or absence of UMI, significant difference in LAD calcium score was noted between those with (n = 16) and without (n = 7) LAD-territorial infarction. LAD calcium score of patients with LAD-territorial infarction was significantly higher than those without it [median (interquartile), 160.5 (25.5–515.5) vs. 7.0 (0.0–18.0), *p =* .034]. In terms of those with UMI of LCX and RCA territories, however, no significant difference in CAC scores of LCX [median (interquartile), 110 (14.5–544.0) vs. 12.5 (0.0–112.3), *p =* .290] and RCA [median (interquartile), 201.0 (51.5–268.5) vs. 61.0 (2.3–385.5), *p =* .507] was observed.

Comparison between those with atypical pattern LGE (n = 12) and without LGE (n = 838) demonstrated no significant difference in baseline characteristics and CMR parameters between two groups.

Among 519 subjects whose risk stratification scores were available, UMI was noted in 12 patients (2.3%). In the comparison between those with and without UMI, subjects with UMI showed higher prevalence of diabetes mellitus (*p* = .041) and higher risk assessment scores such as FRS and ASCVD score compared with those without UMI (*p =* .011 and *p =* .024, respectively). In addition, CAC score was also significantly higher in subjects with UMI (*p <* .001) (Table 2).

**Table 2:** Comparison of risk stratification scores between those with and without late gadolinium enhancement in 519 Subjects.

Parameters	No LGE (n = 507)	UMI (n = 12)	*P value*
Male (%)	464 (91.5%)	12 (100%)	*0*.*611*
Age (years)	53 (50–57)	56.5 (50.5–61.5)	*0*.*246*
BMI (kg/m^2^)	24.7 (23.1–26.5)	26 (22.7–28.0)	*0*.*422*
Hypertension	131 (25.8%)	4 (33.3%)	*0*.*520*
Diabetes Mellitus	57 (11.2%)	4 (33.3%)	***0*.*041***
Hyperlipidemia	126 (24.9%)	5 (41.7%)	*0*.*189*
Family history of CVD	65 (13.5%)	4 (36.4%)	*0*.*054*
Framingham Risk Score	10.5 (7.0–16.6)	17.1 (10.7–31.4)	***0*.*011***
ASCVD Risk Score	5.2 (3.2–8.9)	9.2 (5.3–20.8)	***0*.*024***
CAC Score	3 (0–57.0)	362 (82.3–744.5)	***< 0*.*001***

Note. Median (interquartile range), LGE = late gadolinium enhancement, UMI = unrecognized myocardial infarction, BMI = body mass index, ASCVD = atherosclerotic cardiovascular disease, CAC = coronary artery calcium

### Comparison of UMI prevalence and cohort characteristics according to CAC score

Table 3 demonstrates the result of comparisons according to the CAC score. The prevalence of UMI differed significantly among CAC groups, as 1% in a group without coronary calcium (4/403), 1% in a group of mild calcium (2/293), 6.1% in a group of moderate calcium (7/114) and 14.5% in those with severe coronary calcium (9/62), respectively (*p* < .001). Kruskal-Wallis test and Jonckheere-Terpstra test demonstrated that increasing trend of subjects’ age, BMI, NT-proBNP, plasma glucose level, and LVMI on CMR with increasing amount of coronary artery calcium among CAC groups (*p* < .001, *p* = .022, *p* = .009, *p <* .001, and *p* < .001, respectively). On the other hand, serum cholesterol level and LDL showed negative correlation with significant difference (all *p* = .001), according to CAC groups, which is presumed to be a result of increasing use of statins in those with heavy coronary calcium (*p* = .003).

**Table 3:** Subgroup analysis according to the coronary artery calcium burden.

Parameters	No (CAC = 0) (n = 403)	Mild (1 ≤ CAC < 100) (n = 293)	Moderate (100 ≤ CAC < 400) (n = 114)	Severe (CAC ≥ 400) (n = 62)	*P value*
UMI (%)	4 (1%)	3 (1%)	6 (6.1%)	9 (14.5%)	***< 0*.*001***
Male (%)	363 (90.1%)	281 (95.9%)	112 (98.2%)	61 (98.4%)	***< 0*.*001***
Age (years)	52 (49–55)	53 (50–57)	54 (51–57)	56 (53–64)	***< 0*.*001***
BMI (kg/m^2^)	24.6 (22.9–26.5)	24.7 (23.1–26.4)	25.4 (23.8–27.3)	25.2 (23.5–26.8)	*0*.*022*
NT-proBNP (pg/mL)	20.1 (11.0–34.2)	19.7 (11.5–32.7)	19.9 (11.4–41.1)	34.6.(15.0–52.6)	***0*.*009***
Systolic Blood Pressure (mm Hg)	118 (108.5–130)	119 (112.0–129.8)	118 (110–130)	121 (111–131.5)	*0*.*376*
Diastolic Blood Pressure (mm Hg)	77 (70–84)	78 (72–83)	77 (72–84)	77 (71–84.5)	*0*.*816*
Total cholesterol level (mg/dL)	194 (170.5–221)	199 (169–226)	181 (167–204)	175 (152.5–198.5)	***< 0*.*001***
Triglyceride (mg/dL)	120 (87–171)	120.5 (94.3–174.8)	116 (92–176)	111 (76–148.5)	*0*.*343*
HDL cholesterol level (mg/dL)	52 (45–61)	52 (44–61.8)	52 (43–63.5)	52 (45–63)	*0*.*742*
LDL cholesterol level (mg/dL)	125 (101–148)	129 (103–149.8)	116 (93.5–136)	105 (83–130.5)	***< 0*.*001***
Medication for hyperlipidemia	51 (13.3%)	57 (19.9%)	25 (22.5%)	18 (30%)	***0*.*003***
Plasma Glucose level (mg/dL)	97 (90–104)	98 (92–105)	101 (94.5–112.5)	101 (91–108)	***< 0*.*001***
LVEF, %	66 (52.1–69.4)	66 (62.0–69.8)	67.4 (63.0–71.4)	66.8 (62.3–71.2)	*0*.*169*
LVSV, ml	86.3 (76.0–96.2)	84.8 (75.6–92.5)	88.9 (78.6–99.3)	87.3 (73.6–96.6)	*0*.*163*
Cardiac index, l/min/m^2^	3.1 (2.7–3.4)	3.0 (2.7–3.3)	3.0 (2.8–3.4)	3.0 (2.8–3.3)	*0*.*599*
LVMI, g/m^2^	56.2 (50.8–62.7)	60.1 (53.2–67.2)	61.6 (55.2–69.9)	63.6 (57.4–68.4)	***< 0*.*001***

Note. Median (interquartile range), CAC = coronary artery calcium, UMI = unrecognized myocardial infarction, BMI = body mass index, LVEF = left ventricular ejection fraction, LVSV = left ventricular stroke volume, LVMI = left ventricular mass index

### Comparison of UMI prevalence and cohort characteristics according to FRS

Table 4 demonstrates the comparison of cohort characteristics between low-risk (n = 240), intermediate-risk (n = 204) and high-risk (n = 75) groups, stratified based on 10-year cardiovascular risk of FRS. The prevalence of UMI differed significantly according to the FRS, showing 0.8% in subjects with low risk (2/240), 3% in those with intermediate risk (6/204) and 5.3% in high-risk group (4/75), respectively (*p* = 0.042). The prevalence of hypertension and DM, male-predominance and subjects’ age and BMI were also significantly different according to the FRS (all *p <* .001 and *p =* .001 for BMI, respectively). Risk assessment scores such as ASCVD risk score and CAC score also differed significantly (all *p <* .001). In terms of LV parameters on CMR, LVMI was the only parameter that showed significant difference (*p <* .001).

**Table 4:** Subgroup analysis according to the framingham risk score in 519 subjects.

Parameters	Low (FRS < 10%) (n = 240)	Intermediate (10%≤ FRS< 20%) (n = 204)	High (FRS ≥ 20%) (n = 75)	*P value*
UMI (%)	2 (0.8%)	6 (3.0%)	4 (5.3%)	***0*.*042***
Male (%)	206 (85.8%)	198 (97.0%)	72 (96.0%)	***< 0*.*001***
Age (years)	52 (49–55)	55 (51–58)	60 (53–66)	***< 0*.*001***
BMI (kg/m^2^)	24.4 (22.7–26.2)	25.1 (23.5–27.0)	24.9 (23.7–26.8)	***0*.*001***
Hypertension	36 (15.0%)	68 (33.3%)	31 (41.3%)	***< 0*.*001***
Diabetes Mellitus	10 (4.2%)	26 (12.7%)	25 (33.3%)	***< 0*.*001***
Hyperlipidemia	50 (20.8%)	58 (28.4%)	23 (30.7%)	*0*.*093*
Family history of CVD	29 (12.6%)	28 (14.6%)	12 (16.7%)	*0*.*654*
NT-proBNP (pg/mL)	20.0 (11.5–35.4)	20.3 (11.2–34.5)	27.9 (13.8–47.8)	***0*.*034***
ASCVD Risk Score	3 (2.3–4.1)	7.3 (5.8–9.2)	15.4 (12.5–21.4)	***< 0*.*001***
CAC Score	0 (0–31)	11 (0–81.8)	27 (0–147)	***< 0*.*001***
LVEF, %	66.4 (62.3–70.1)	66.1 (61.9–69.6)	67.9 (63–72.8)	*0*.*069*
LVSV, ml	86 (76.6–96.8)	88.5 (76.7–96.8)	87.8 (78.7–100.2)	*0*.*649*
Cardiac index, l/min/m^2^	3.1 (2.8–3.4)	3.0 (2.7–3.3)	3.1 (2.8–3.4)	*0*.*193*
LVMI, g/m^2^	55.0 (49.7–62.1)	67.0 (55.0–67.5)	62.1 (54.0–69.7)	***< 0*.*001***

Note. Median (interquartile range), UMI = unrecognized myocardial infarction, BMI = body mass index, CVD = cardiovascular disease, ASCVD = atherosclerotic cardiovascular disease, CAC = coronary artery calcium, LVEF = left ventricular ejection fraction, LVSV = left ventricular stroke volume, LVMI = left ventricular mass index

### Diagnostic accuracy for predicting UMI in asymptomatic subjects

The ROC curve by using CAC score in 872 subjects demonstrated an area under the ROC curve (AUC) of 0.795 (95% confidence interval (CI), 0.766–0.821; *p* < .0001) with a cutoff value of 79, showing a sensitivity of 78.3% and a specificity of 78.5%. For 519 patients, the AUCs for CAC score, FRS and ASCVD risk score were 0.816 [95% CI, 0.780–0.848; *p* < .001] with a cutoff value of 79, 0.712 [95% CI, 0.671–0.751; *p* = .009] with a cutoff value of 15.8 and 0.689 [95% CI, 0.648–0.729; *p* = .036] with a cutoff value of 17.2, respectively.

As CAC scoring CT is generally indicated for intermediate-risk subjects according to the 2010 guideline for the appropriate use of cardiac CT [20], we additionally performed subgroup ROC analysis for 204 patients of intermediate-risk group. The rate of UMI in intermediate-risk group was 2.9% (6 of 204). The AUCs of FRS and ASCVD risk score were as low as 0.552 (95% CI, 0.481–0.622; *p* = .723) and 0.526 (95% CI, 0.455–0.596; *p* = .856) without statistical significance for predicting UMI. However, the AUC for CAC score was 0.780 (95% CI, 0.717–0.835; *p* = .020) with a cutoff value of 77, showing a sensitivity of 83.3% and a specificity of 75.3%.

## Discussion

In this study, we have demonstrated that CAC score is a good discriminator for UMI, superior to FRS and ASCVD risk scores, in asymptomatic Asian cohort. Furthermore, our ROC analyses suggest a potential role of CAC score as a predictor of UMI in those with intermediate risk of cardiovascular disease, as indicated in the 2010 guideline by ACCF/SCCT/ACR/AHA/ASE/ASNC/NASCI/SCAI/SCMR.[20] We also demonstrated that the prevalence of UMI is significantly higher with increasing burden of coronary artery calcium and increasing risk of 10-year cardiovascular disease based on FRS in subgroup analyses. These results are consistent with previous reports which have suggested that CAC score is a sensitive, accurate, and reproducible parameter, reflecting coronary atherosclerosis.[21,22] In addition, prior study has shown that CAC score predicts cardiac death, nonfatal MI, and the need for coronary revascularization in asymptomatic patients.[23] It may be hard to generalize our result due to relatively low event rate of UMI, however, our study has clinical implication that it shows a potential of CAC score as a predictor of UMI in asymptomatic general population as well as for those indicated according to the guideline.

One of unique result of our study is that subjects with UMI did not show any functional degradation, such as LVEF and cardiac index, compared with those without UMI. Many of prior studies, on the contrary, have stated a prognostic implication of UMI, demonstrating impaired LV function and increased myocardial mass in those with UMI compared with normal groups.[3,6,8,24] One of the reasons for this conflicting result can be a younger age of our study population (53.88 ± 5.91), compared with prior studies with a mean age of 69 years and 65.4 years, respectively.[3,8] J.R.Weir-McCall et al. had reported that UMIs had significant functional implications in relatively young age group with a mean age of 54 years, however, the incidence of UMI was extremely low (0.2%; 3/1529), making it hard to generalize the results.[6] Our study may explain the reason why these MIs were remained “silent” and “unrecognized”. Considering 15 of 23 UMIs were focal infarction involving only one segment and 19 of 23 UMIs were non-tranmural (subendocardial) infarctions, the lesions might not be critical enough to result in chest pain and might be easily compensated without cardiac dysfunction, unlike recognized MI. Though we did not deal with MACE and cardiac mortality as a final outcome due to relatively short follow up period, UMI may not always be a decisive factor for poor prognosis. Long-term study based on multi-ethnic group with large population is required to determine the real clinical consequences of UMI, in terms of cardiac function and prognosis.

CMR is a valuable modality in the detection of UMI. Prior studies have shown that CMR can detect UMIs with a higher sensitivity, specificity and reproducibility than ECG.[24,25] Indeed, only three of 23 subjects with UMI in our study demonstrated abnormal ECG, such as pathologic Q wave or ST-segment changes. Hence, we cannot regard ECG as a stand-alone screening modality for MI and CMR should be strongly recommended for those with high risk of MI.

In this study, the prevalence of UMIs in asymptomatic Asian subjects was 2.3%. This is far lower rate of UMI compared with Sweden and Iceland cohorts, described a prevalence of 30% and 16.8%, respectively.[4,26] Main reason of this discrepancy is that these prior studies had focused on an older age group with a mean age of 75 years compared with the current study of 54.5 years, showing a strong association between UMI and age. Meanwhile, US cohort and Scottish cohort, composed of middle-aged population with an intermediate or low cardiovascular risk, demonstrated very low rate of UMIs of 0.2%, suggesting that high risk group of cardiovascular disease is strongly associated with UMIs.[3,6] Our study population is consisted of asymptomatic self-referred Asians without prior hospital-reported ischemic heart disease. We did not set any special requirements such as age or cardiovascular risk factors (i.e., hypertension, diabetes mellitus, or hyperlipidemia) in the study enrollment. Although it is hard to generalize our findings in all of the other groups, we believe that the prevalence of UMIs of our cohort might be closer to that of general population.

Atypical LGE, not typical MI, was noted in 12 of 872 patients, and the most common finding was linear mid-wall enhancement. These atypical LGE is known to be seen in infiltrative myocardial disease or myocarditis, however, there was no significant difference in baseline characteristics and CMR parameters between those with atypical LGE and normal group in our study.[27] Although the exact pathophysiology of atypical myocardial scar is still unknown, there have been prior reports on the atypical LGE, not MI, on CMR.[6] We think that it should be an another area of investigation whether these atypical myocardial scars are a distinct disease entity or just a normal variation of LV myocardium.

There are several limitations in our study. First, our study was limited inherently by its retrospective design. Second, our study was performed at a single health promotion center of tertiary referral hospital. All of the subjects in our study were self-referred for a routine health check-up, who were predominantly male, and therefore, selection bias may limit the generalizability to other populations. Third, cardiovascular risk scores such as FRS and ASCVD score were available only in 519 of 872 subjects due to lack of medication history of hypertension. Fourth, the prevalence of UMI was low, resulting in small sample size and limited power for analysis. However, we tried to identify the incidence of UMI among asymptomatic Asian population in this study. Fifth, tiny high signal intensity might be missed if placed in between two consecutive slices of LGE imaging, which can underestimated the prevalence of UMI.

In conclusion, our study demonstrated that the prevalence of UMI increases with increasing burden of CAC and increasing risk of 10-year cardiovascular disease on FRS. CAC score is a good discriminator for UMI, superior to FRS and ASCVD risk scores, in asymptomatic Asian population, especially for those with intermediate risk of cardiovascular disease, as indicated in 2010 guideline for cardiac CT. Furthermore, subjects with UMI did not show any LV functional impairment compared with those without UMI in our study, thus, further study to figure out the final consequences of UMI in general population, regarding cardiac function and prognosis, can be a next step.
